# Detection of Abrin Holotoxin Using Novel Monoclonal Antibodies

**DOI:** 10.3390/toxins9120386

**Published:** 2017-11-28

**Authors:** Xiaohua He, Stephanie Patfield, Luisa W. Cheng, Larry H. Stanker, Reuven Rasooly, Thomas A. McKeon, Yuzhu Zhang, David L. Brandon

**Affiliations:** Western Regional Research Center, U.S. Department of Agriculture, Agricultural Research Service, 800 Buchanan Street, Albany, CA 94710, USA; stephanie.patfield@ars.usda.gov (S.P.); luisa.cheng@ars.usda.gov (L.W.C.); larry.stanker@ars.usda.gov (L.H.S.); reuven.rasooly@ars.usda.gov (R.R.); thomas.mckeon@ars.usda.gov (T.A.M.); Yuzhu.zhang@ars.usda.gov (Y.Z); david.brandon@ars.usda.gov (D.L.B.)

**Keywords:** abrin, agglutinin, enzyme-linked immunosorbent assay, milk, monoclonal antibody, ricin, Western blot

## Abstract

Abrin, a member of the ribosome-inactivating protein family, is produced by the *Abrus precatorius* plant. Having the potential to pose a severe threat to both human and animal health, abrin is classified as a Select Agent by the U.S. Department of Health and Human Services. However, an immunoassay that is specific for intact abrin holotoxin has not yet been reported. In this study, seven new monoclonal antibodies (mAbs), designated as Abrin-1 through Abrin-7 have been developed. Isotyping analyses indicate these mAbs have IgG1, IgG2a, or IgG2b heavy-chains and kappa light-chains. Western blot analyses identified two abrin A-chain specific mAbs, Abrin-1 and Abrin-2, and four B-chain specific mAbs (Abrin-3, -5, -6, and -7). A sandwich enzyme-linked immunosorbent assay (ELISA), capable of detecting a mixture of abrin isoforms and agglutinins was developed using B-chain specific Abrin-3 for capture and A-chain specific Abrin-2 as detector. The ELISA is highly sensitive and detects 1 ng/mL of the abrin holotoxin in phosphate-buffered saline, nonfat milk, and whole milk, significantly below concentrations that would pose a health concern for consumers. This ELISA also detects native abrin in plant extracts with a very low background signal. The new abrin mAbs and ELISA should be useful for detecting this potent toxin in the milk supply chain and other complex matrices.

## 1. Introduction

The rosary pea, *Abrus precatorius*, is native to India and grown in tropical and subtropical areas of the world. It is extremely invasive, and its deep roots are very difficult to remove once it has been established under favorable conditions. The plant is best known for its brightly colored and hard-shelled seeds, which are sometimes used as beads for necklaces, bracelets, and percussion instruments. When the shell is damaged, the seed becomes very toxic due to the presence of abrin, one of the most poisonous naturally occurring plant toxins. Abrin is synthesized as a single protein consisting of a 34-amino acid leader sequence, an A-chain of 251 amino acids, a 14-amino acid linker, and a B-chain of 263 amino acids [1]. The A-chain and B-chain are linked by a single disulfide bond to form a holotoxin (65 kDa) [2]. Abrin A-chain (~28 kDa) is an *N*-glycosidase that depurinates the 28S rRNA of eukaryotes at the position A4324, preventing the binding of ribosomes to elongation factors and thereby inhibiting protein synthesis, resulting in cell death [3,4]. Abrin B-chain (~30 kDa) is a lectin that binds to host cell surface receptors containing galactose and carries the whole toxin into the cells through endocytosis [5]. The reduction of the A-B inter-subunit disulfide bond is essential for the cytotoxicity once the toxin enters the cells [6]. Along with four isoforms of abrin, abrin-a, -b, -c, and -d, one agglutinin (a hemagglutinin) has been isolated from the seeds of *Abrus precatorius* [7]. The four abrin isotoxins have similar amino acid composition, but different cytotoxicity. The LD_50_ values determined through intraperitoneal (ip) injection of Swiss white mice were 10, 25, 16, and 31 µg/kg body weight for abrin-a, b, c, and d, respectively [7]. *Abrus* agglutinin is a heterotetramer (~120 kDa), made up of two A and two B chains [6]. Although highly homologous in sequence with abrin, the agglutinin is 200- to 2000-fold weaker in cytotoxicity, a characteristic attributed to the substitution of Asn-200 in abrin with Pro-199 in agglutinin [6]. *Abrus* agglutinin is a strong hemagglutinin, agglutinating human red blood cells at concentrations as low as 30 ng/mL [6].

Abrin is very similar to another plant toxin, ricin, produced by castor beans, in protein sequence and mechanism of toxin action [8,9]. However, the estimated fatal human dose of abrin is 0.1–1 µg/kg via intravenous injection (iv), less than half that of ricin [10]. Due to its strong inhibitory activity on eukaryotic ribosomes that leads to cell death, abrin has been frequently reported as an immunotoxin for the treatment of cancer [11]. However, its high toxicity and ease of production from natural sources makes abrin a potential threat agent. Abrin seeds have resulted in accidental poisoning of young children following ingestion due to their colorful appearance, and they have also been used in attempted suicides [12,13]. To reduce morbidity and possible mortality, it is critical to develop sensitive methods for detection of abrin. Abrin can be detected by its activity, or structure, or by the presence of surrogate compounds associated with the seed. A recent review summarizes principal detection methodologies [14]. Both enzyme-linked immunosorbent assay (ELISA) and lateral flow methods have been reported for abrin with a limit of detection (LOD) between 0.1 and 0.5 ng/mL [15,16], but sensitive assays that specifically detect abrin holotoxins and not the isolated A and B chains are still lacking in the literature.

In this study, we report the development and characterization of 7 novel monoclonal antibodies (mAbs) against abrin. Two mAbs with different chain specificity were selected and used to assemble a sandwich ELISA, which was shown to be specific for intact holotoxin that contains both A- and B-chains. Due to the great concern over the possibility of terrorist poisoning of thousands of people through the milk supply [17], milk was chosen as the first food matrix to test the assay practicality. In addition, this ELISA was also tested for its ability to detect abrin in crude plant extracts.

## 2. Results

### 2.1. Development and Characterization of Monoclonal Antibodies against Abrin

To identify mAbs against abrin, a total of 960 wells of splenocyte-myeloma hybridoma culture were screened for abrin recognition. Positive signals (signal to noise ratio of five or greater) were observed from 50 of the hybridoma culture supernatants. The cells from these wells were expanded, tested and cloned by limiting dilution to produce hybridoma cell lines. After repeated cloning and recovery steps, seven mAbs, designated Abrin-1 through Abrin-7, were selected for further characterization because of their strong binding under standard assay conditions. Table 1 summarizes the isotype and subunit specificity of these mAbs. Antibody isotype was determined by ELISA using abrin-coated microtiter plates and horseradish peroxidase-conjugated, isotype-specific antibodies. The ELISA results demonstrated that three mAbs (Abrin-1, Abrin-2, and Abrin-7) possess gamma-2a heavy chains, two mAbs (Abrin-3, Abrin-5) have gamma-2b, and two mAbs (Abrin-4, Abrin-6) have gamma-1 heavy chains. All mAbs possess kappa light-chains.

**Figure 1:**
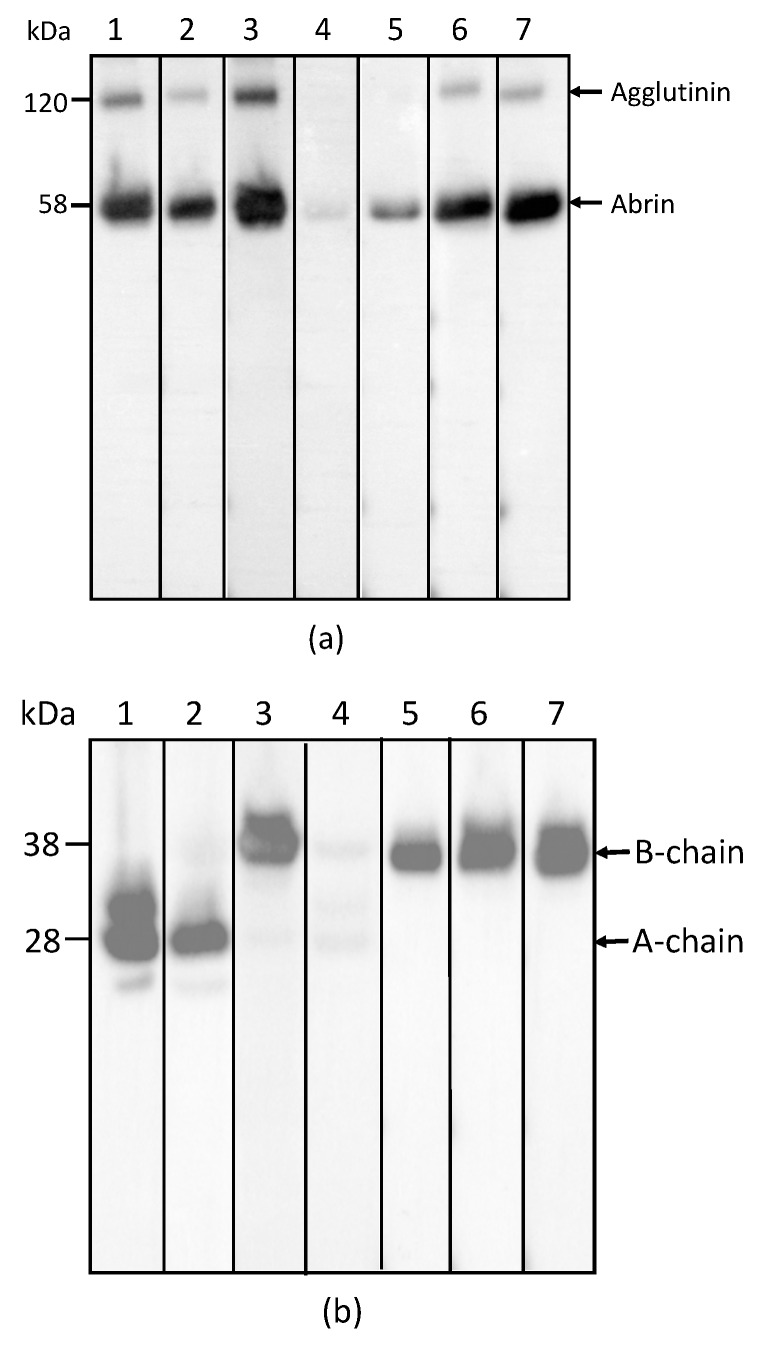

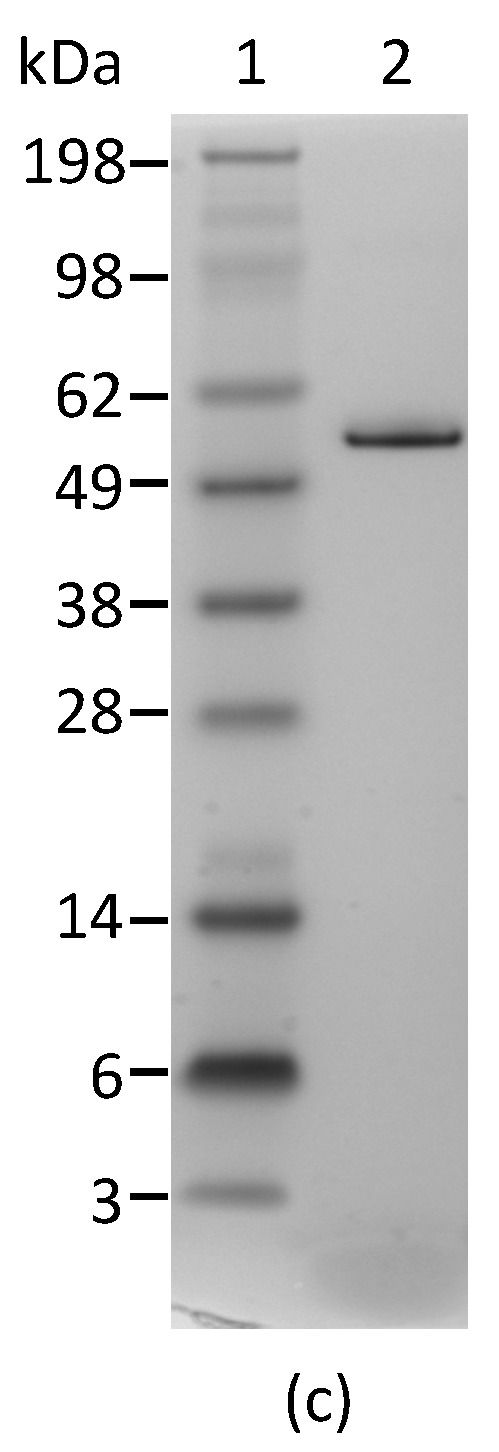
Reactivity of mAbs to abrin and agglutinin determined by Western blot analysis. Each lane was loaded with 250 ng of the abrin preparation and separated by SDS-PAGE under non-reducing (**a**) and reducing (**b**) conditions. Membranes 1–7 were incubated with mAbs, Abrin-1, to -7, respectively. The sizes of the agglutinin and abrin are indicated in kilodaltons (kDa) at the left side of panel (**a**) and the sizes of the A-chain and B-chain of the abrin/agglutinin are indicated at the left side of panel (**b**). The purity of the abrin (1 µg) used in this study was examined by non-reducing SDS-PAGE, followed with SimpleBlue staining (**c**).

In order to determine the specificity of each mAb for abrin and agglutinin, the abrin preparation purified from rosary peas and containing both abrin and agglutinin were analyzed by Western blot following SDS-PAGE under non-reducing conditions. Figure 1a indicates that mAbs Abrin-1, -2, -3, -6, and -7 are able to bind both abrin and agglutinin. The blots with mAbs Abrin-4 and -5 show binding to abrin, albeit with a weak signal, but no binding to the agglutinin based on the Western blot analysis results. Although the strength of the blot signal varied among the seven mAbs tested, the basis of this variation (e.g., mAb affinity, the nature and stability of the epitope recognized) was not investigated in this study. To determine the chain-specificity of each mAb, Western blot analyses were performed following SDS-PAGE under reducing condition. Figure 1b demonstrates that mAbs Abrin-1, Abrin-2 bind to the A-chain, while mAbs Abrin-3, Abrin-5, Abrin-6, and Abrin-7 bind to the B-chain. The mAb Abrin-4 seems to bind both A- and B-chains, but very weakly, therefore, the chain-specificity is listed as not determined (nd) in Table 1. It is possible that mAb Abrin-4 does not bind well to denatured abrin in SDS-PAGE, although it was selected based on its strong binding to abrin pre-coated in 96-well plates. All antibodies were screened by direct ELISA, in which supernatants from hybridoma cell cultures were added to plates pre-coated with antigen. In addition, mAb Abrin-1 reacts to an unknown protein above the A-chain under reducing condition. To examine the quality of the abrin used in this study, non-reducing SDS-PAGE of the abrin was performed followed by staining with SimplyBlue (Figure 1c). No visible agglutinin or other contaminants were observed, suggesting the toxin preparation was pretty clean, and the amount of agglutinin in the toxin preparation was much less than abrin. This is consistent with the result shown in Figure 1a. None of the seven abrin mAbs cross-reacted with ricin when tested by Western blot (data not shown).

### 2.2. Establishment of an ELISA that Detects Abrin Holotoxin

**Figure 2:**
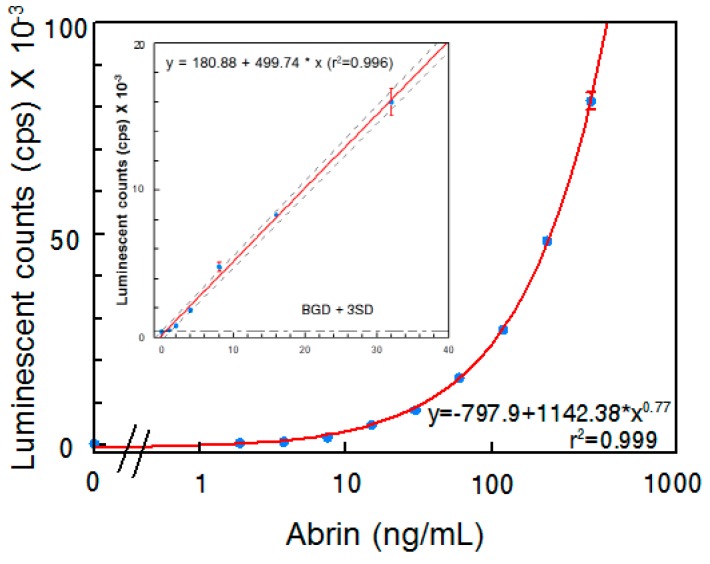
Detection of abrin in PBS by ELISA. Data represent the average of three determinations ± SD. Limit of detection (LOD) for the mixture of abrin was 1 ng/mL. The one-sided 95% confidence intervals on the fitted line are shown as dashed curves.

Although several abrin ELISAs have been reported in the literature [15,18,19], an ELISA that requires both A- and B-chains be present has not been reported. In an attempt to develop a sandwich ELISA that selectively detects active abrin, detection of both A- and B-chains seemed desirable. Each of the two abrin A-chain specific mAbs were paired with each one of the four B-chain-specific mAbs developed in this study and evaluated for their performance as capture or detector antibodies. The most sensitive ELISA was obtained when using mAb Abrin-3 for capture and biotinylated mAb Abrin-2 as a detector. Figure 2 illustrates the results obtained using this sandwich ELISA to detect the abrin preparation in phosphate-buffered saline (PBS). The standard curve has a slight sigmoidicity as is usual for ELISA, but the linear regression within the range of 0–32 ng/mL has a *R*^2^ > 0.99. Therefore, for samples with abrin concentrations higher than 32 ng/mL, appropriate dilutions are needed for accurate quantification. The average background signal was 395 ± 7 and the LOD is 1 ng/mL.

### 2.3. Detection of Abrin Produced by Plants and Spiked in Milk

To investigate the utility of this ELISA for detection of native abrin produced by rosary pea, crude extracts were prepared. Table 2 exhibits ELISA results for abrin determined based on the ELISA signals (counts per second or cps). The ELISA signal for abrin in rosary pea extract was surprisingly high, even in 0.02% rosary pea extract (43,285 cps) and increased with the increase of plant extract concentration. In contrast, the ELISA signals for castor bean extract were between 150 and 210 cps, not significantly different from the background signal of the PBS control. The low background was obtained even when the plant extract concentration was increased to 2%, suggesting this ELISA is not impacted by cross-reactivity with components of castor bean extracts and is highly selective for abrin produced by rosary pea. The selectivity is notable because ricin is highly similar to abrin and was present in the castor bean extract at almost 5% [20].

**Figure 3:**
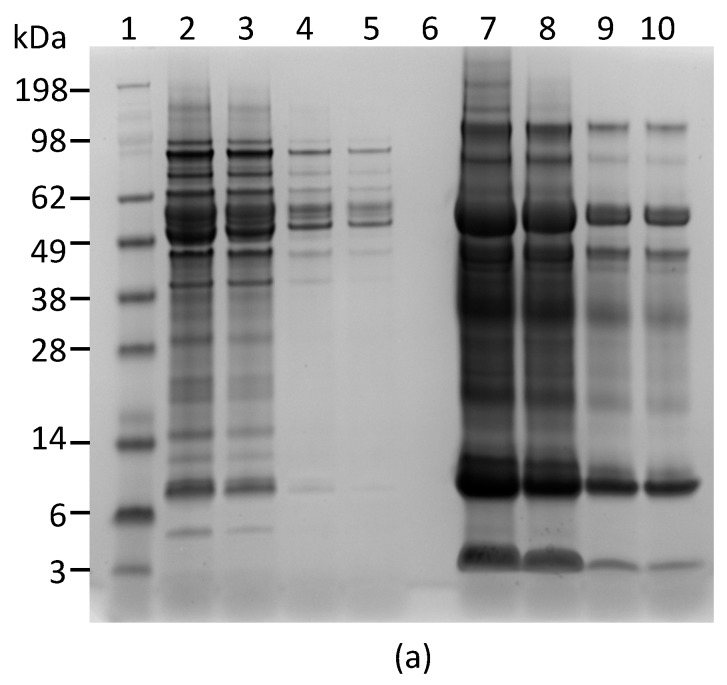

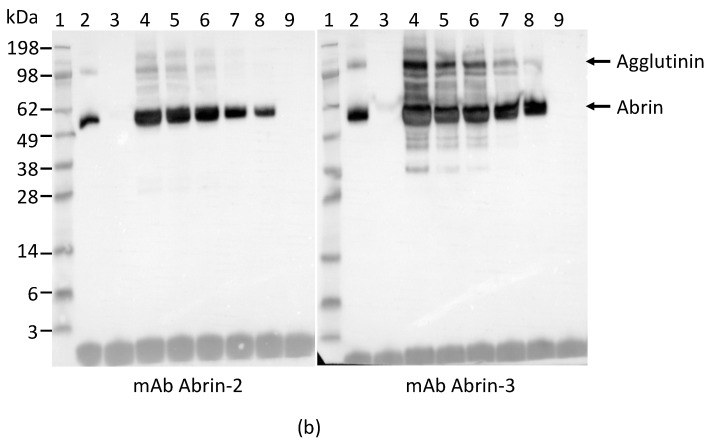
Reactivity of mAb Abrin-2 and Abrin-3 to extracts of rosary pea and castor bean. (**a**) Analysis of extracts by SDS-PAGE stained with SimpleBlue: Lane 1, protein markers; Lanes 2–5 were loaded with 10, 5, 1, and 0.5 µL of 2% rosary pea extract; Lane 6, blank; Lanes 7–10 were loaded with 10, 5, 1, and 0.5 µL of castor bean extract. (**b**) Western blot analysis: Lane 1, protein markers; Lane 2, 0.1 µg of purified abrin; Lane 3, blank; Lanes 4–8 were loaded with 10, 5, 3, 1, and 0.5 µL of 2% rosary pea extract; Lane 9, 10 µL of 2% castor bean extract. Proteins were separated by SDS-PAGE under non-reducing condition. The expected sizes of the agglutinin and abrin are indicated at the right side of the blots.

We also performed Western blot analysis to confirm that both capture and detection antibodies used in the ELISA react with abrin produced by rosary peas. Figure 3a shows the SDS-PAGE of the plant extracts. Figure 3b indicates that the mAbs Abrin-2 and Abrin-3 only bind to proteins with predicted MW for abrin and agglutinin in rosary pea extract, but not to any proteins in castor bean extract.

**Figure 4:**
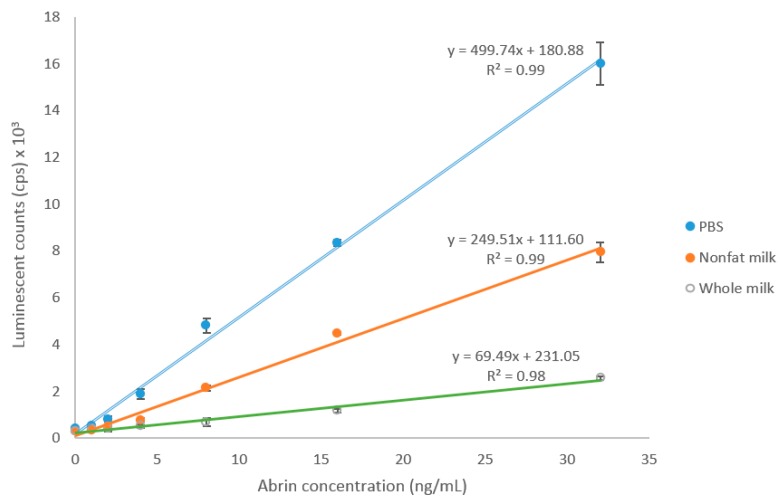
ELISA detection of abrin spiked in milk. Data shown are average ELISA readings (cps) ± SD, *n* = 6.

To investigate the feasibility of using this ELISA to detect abrin contamination in milk, undiluted nonfat and whole milk (1 mL) were spiked with various amounts of abrin in 10 µL of PBS and analyzed by ELISA. Results from the assays indicate that milk significantly affects the ELISA for abrin detection, suppressing the response and increasing the background signal. The matrix effect from whole milk is bigger than that from nonfat milk based on the bigger slope difference. In spite of the low recovery rate, the LOD for abrin in nonfat and whole milk is still 1 ng/mL (Figure 4).

## 3. Discussion

Abrin has been exploited as a biological weapon and an agent of bioterrorism and has been classified as a Select Agent by the Centers for Disease Control and Prevention and the Animal and Plant Health Inspection Service [21]. However, reagents for immunoassays of this potent and widely available toxin are still very limited compared to those for ricin. ELISAs developed by Garber et al. in 2008 [15] and Zhou et al. in 2012 [19] were successfully validated for detection of abrin in various food matrices. In their assays, a combination of monoclonal/polyclonal antibody or polyclonal/monoclonal antibody was used for capture and detection. Both assays were highly sensitive, but they were not designed for exclusive detection of intact or active abrin due to the nature of antibodies used. A llama-derived single domain antibody based ELISA reported in 2011 by Goldman et al. [22] detected *Abrus* agglutinin, exclusively, leaving all abrin isoforms undetected. Assays that fail to detect major abrin isoforms could yield misleading diagnostic results, with a consequent risk to human health. Like many other toxic lectins in the AB family of toxins, abrin consists of two functionally different parts, an enzymatically active A-chain and a receptor-binding B-chain. Although it is enzymatically active, free A-chain is non-toxic to intact cells because it lacks the ability to bind to and enter cells in the absence of B-chain. Both chains are required for abrin cytotoxicity [23]. In this study, we sought to develop mAbs with special characteristics that would enhance our capability to detect active abrin. Two A-chain specific and four B-chain specific monoclonal antibodies were isolated and characterized. Monoclonal antibodies, Abrin-2 and Abrin-3, had different chain specificity when analyzed by Western blot (Figure 1b) and exhibited the greatest compatibility among all combinations of mAbs tested in sandwich ELISAs. Therefore, this pair was selected, with Abrin-3 as capture antibody and Abrin-2 as detection antibody. This ELISA was demonstrated to be very sensitive and the LOD for abrin was 1 ng/mL in milk samples. The abrin used in this study was a commercially available mixture of abrin isoforms and agglutinin. Currently, commercial standards for abrin isoforms and agglutinin are not available. To enable us to test the ELISA for detection of each individual abrin isoform and agglutinin, the development of non-toxic recombinant toxoids of abrin isoforms and agglutinin is underway in our laboratory. These reagents could then be used as standards or as antigens for developing new antibodies and abrin isoform- and agglutinin-specific immunoassays. Such assays will be useful to evaluate both the composition and toxicity of samples such as “white powders” encountered in defense against bioterrorism because the abrin isoforms and the agglutinin differ in toxicity.

The overall goal for this study is to develop an assay for detecting intentional adulteration of foods with abrin. Since it is likely that a crude, rather than highly purified preparation of abrin would be used as a bioweapon, it is important to detect abrin as it occurs in crude extracts. In order to assess potential interfering substances in the crude extract, an ideal negative control would be prepared from rosary peas that lack abrin. However, abrin-negative rosary peas are unavailable. Therefore, castor bean extracts that contain the analogous protein, ricin, were used as negative controls. Results from ELISA cross-reactivity tests demonstrated that the new ELISA produced positive responses only with rosary pea extracts, not with castor bean extracts (Table 2). Thus, crude seed extracts do not elicit false-positive signals in the ELISA. Furthermore, Western blot analysis showed that the two mAbs used in the ELISA bound to the abrin present in the rosary pea extract, but not to any proteins in the castor bean extract (Figure 3). These results support the conclusion that the ELISA is specific for abrin.

The milk industry is a vital component of food production worldwide, as well as an important contributor to the economies of modern nations. For example, cash receipts from the marketing of milk in the USA totaled $34.5 billion in 2016 [24]. However, the milk industry could be a vulnerable target to the bioterrorist, without sufficient security and safety preparedness. Therefore, we validated the new assay for use in case of a deliberate act of adulteration by testing undiluted nonfat and whole milk samples that had been spiked with abrin. The assay sensitivity enables detection of 1 ng/mL or less than 0.25 µg of abrin in a typical one cup serving of milk. This level is much lower than 350–490 µg, the oral toxic dose for a 70 kg adult (5–7 µg/kg). On the basis of the results presented, the new mAbs and ELISA developed in this study could be valuable tools for detecting the adulteration of milk products with abrin.

## 4. Materials and Methods

### 4.1. Ethics Statement

All procedures with animals were carried out according to institutional guidelines for husbandry (USDA ARS Directive #130.4.v.3, 17 July 2013) and specific procedures and protocols for antibody production were reviewed and approved by the Western Regional Research Center (WRRC) Institutional Animal Care and Use Committee (IACUC). Mice were euthanized using rapid cervical dislocation.

### 4.2. Monoclonal Antibody Production and Purification

Abrin (a mixture of abrin isoforms and agglutinin) was purchased from Toxin Technology (Sarasota, FL, USA). Abrin toxoid was prepared by heating abrin at 70 °C for 30 min. Monoclonal antibodies were produced using protocols described, previously [25] with minor modifications. Briefly, heat-inactivated abrin was used as an immunogen and injected into female BALB/c mice via intraperitoneal injection (Simonsen Laboratory, Gilroy, CA, USA) at two-week intervals for a total of three injections. Two weeks after the third injection, mice were boosted intravenously with 1 µg of abrin toxoid in sterile PBS. Four days later, mice were euthanized by rapid cervical dislocation, spleens were excised aseptically, and splenocytes were harvested. SP2/0 myeloma cell and splenocyte cell fusions were achieved using polyethylene glycol [26]. Following cell fusion, supernatants of cell cultures were screened using direct ELISA by adding supernatants to plates pre-coated with abrin. After adding goat anti-mouse secondary antibody, the hybridomas were evaluated based on the ELISA signals. Hybridoma cell lines that produced abrin-binding antibodies were cloned by limiting dilution, regrowth, and screening (typically three rounds of cloning). Antibodies were purified by affinity chromatography on Protein-G-conjugated Sepharose (Sigma, St. Louis, MO, USA). Protein concentrations were determined with the BCA Protein Assay Kit (Pierce, Rockford, IL, USA). Biotinylation of antibodies was performed using a Lightning-Link Biotin Conjugation Kit (Innova Biosciences, Cambridge, UK). Antibody isotype was determined by ELISA using toxin-coated microtiter plates and horseradish peroxidase-conjugated, isotype-specific antibodies (SouthernBiotech, Birmingham, AL, USA).

### 4.3. Enzyme-Linked Immunosorbent Assay (ELISA)

Sandwich ELISAs were performed as described previously with slight modification [25]. Briefly, plates were coated with a selected capture mAb (5 µg/mL, 100 µL/well) in PBS and incubated overnight at 4 °C. Plates were then blocked by adding 300 µL of 3% bovine serum albumin (BSA) in Tris-buffered saline with 0.05% Tween-20 (TBST) and incubating for 1 h at room temperature (RT). After brief washing, abrin samples were added (100 µL) to each well and the plates were incubated for 1 h at RT. The plates were washed six times with TBST. Next, biotinylated detection mAb (100 µL/well of 200 ng/mL in 3% BSA-TBST) was added. The plates were incubated for 1 h at RT then washed six times with TBST. One hundred µL/well of 1:10,000 dilution of streptavidin-HRP (Invitrogen, Carlsbad, CA, USA) in 3% BSA-TBST were added and then incubated for 1 h at RT. After a final washing, SuperSignal West Pico Chemiluminescent Substrate (Pierce) was added (100 µL/well). Luminescence was measured in counts per second (cps) using a Victor 3 plate reader (Perkin-Elmer, Shelton, CT, USA). ELISAs were conducted at least three times for confirmation. The limit of detection was determined as the lowest toxin concentration of analyte that generated a response greater than the background plus three times the standard deviation.

### 4.4. Polyacrylamide Gel Electrophoresis (PAGE) and Western Blot Analyses

All gel electrophoresis equipment, buffers, gels and PVDF membranes were purchased from Invitrogen (Carlsbad, CA, USA). Pure or crude abrin samples were denatured at 72 °C for 10 min in NuPage LDS loading buffer, and then separated on a 4–12% NuPAGE Novex Bis-Tris mini gel. To visualize proteins directly after gel electrophoreses, gels were stained with SimplyBlue SafeStain (ThermoFisher, Waltham, MA, USA). For Western blot analyses, proteins were transferred to a PVDF membrane (pore size, 0.45 μm), blocked with 2% ECL Prime blocking agent (GE Healthcare) in PBST, and washed 3 times with PBST. Monoclonal antibodies were diluted to 200 ng/mL in blocking solution and incubated with the blots for 1 h at RT. After washing (3 times) in PBST, the blots were incubated with horseradish peroxidase-conjugated goat anti-mouse (GAM-HRP) antibody (Promega, Madison, WI, USA) at 25 ng/mL for 1 h at room temperature (RT). The blots were developed using TMA-6 substrate (Lumigen, Southfield, MI, USA) and visualized using a G:Box Chemi xx6 (Syngene, Cambridge, UK). All Western blots were replicated at least three times.

### 4.5. Preparation of Plant Seed Extracts

Dry castor seeds and rosary peas with shell removed were ground into <1 mm meal with a Thomas Wiley Mini-Mill (Thomas Scientific, Swedesboro, NJ, USA). Seed meals were extracted with acetone (5 mL/g of seed meal) two times to remove seed oil, and the resulting paste was spread on a sheet of paper in a fume hood to air dry at room temperature overnight. Crude abrin and ricin extracts were prepared by homogenizing oil-free meals in PBS (1 g meal in 10 mL of PBS) with a mortar and pestle. The aqueous extract was filtered using a 0.45 μm filter to remove particulates.

## Figures and Tables

**Table 1:** Some characteristics of abrin monoclonal antibodies.

Antibody	Isotype	Subunit Specificity
Abrin-1	IgG2a, kappa	A
Abrin-2	IgG2a, kappa	A
Abrin-3	IgG2b, kappa	B
Abrin-4	IgG1, kappa	Nd *
Abrin-5	IgG2b, kappa	B
Abrin-6	IgG1, kappa	B
Abrin-7	IgG2a, kappa	B

* not determined.

**Table 2:** ELISA reactivity with plant extracts at three concentrations.

Plant	Extract (wt/vol)	Average ELISA Reading (cps)	SD
Rosary pea	0.02%	43,285	3155
	0.20%	74,465	2335
	2.00%	98,690	930
Castor bean	0.02%	160	10
	0.20%	210	30
	2.00%	150	30
PBS	0	227	30

Data represent the average of three determinations. SD, standard deviation.
